# rtM204Q May Serve as a Novel Lamivudine-Resistance-Associated Mutation of Hepatitis B Virus

**DOI:** 10.1371/journal.pone.0089015

**Published:** 2014-02-24

**Authors:** Yan Liu, Zhihui Xu, Yan Wang, Xiaodong Li, Liming Liu, Li Chen, Shaojie Xin, Dongping Xu

**Affiliations:** Institute of Infectious Diseases/Liver Failure Medical Center, Beijing 302 Hospital, Beijing, China; Institut Pasteur, France

## Abstract

**Background and Aims:**

Lamivudine (LAM) is still widely used for anti-HBV therapy in China. The study aimed to clarify whether a newly-found rtM204Q mutation from patients was associated with the drug resistance.

**Methods:**

HBV complete reverse-transcriptase region was screened by direct sequencing and verified by clonal sequencing. Replication-competent plasmids containing patient-derived 1.1mer mutant or wild-type viral genome were constructed and transfected into HepG2 cells. After cultured with or without serially-diluted antiviral drugs, intracellular HBV replicative intermediates were quantitated for calculating the 50% effective concentration of drug (EC_50_).

**Results:**

A total of 12,000 serum samples of 9,830 patients with chronic HBV infection were screened. rtM204Q mutation was detected in seven LAM-refractory patients. By contrast, rtM204I/rtM204V mutations were detected in 2,502 patients' samples. The rtM204Q emerged either alone or in concomitance with rtM204I/rtM204V, and all were accompanied with virologic breakthrough in clinical course. Clonal sequencing verified that rtM204Q mutant was predominant in viral quasispecies of these samples. Phenotypic analysis showed that rtM204Q mutant had 89.9% of replication capacity and 76-fold increased LAM EC_50_ of the concomitant wild-type strain. By contrast, rtM204I mutant in the sample had lower replication capacity and higher LAM resistance (46.3% and 1396-fold increased LAM EC_50_ of the wild-type strain) compared to rtM204Q mutant. rtM204Q mutant was susceptible to adefovir dipivoxil (ADV) *in vitro* and ADV/ADV+LAM rescue therapy in clinic.

**Conclusion:**

rtM204Q is suggested to be a novel LAM-resistance-associated mutation. It conferred a moderate resistance with higher competent natural replication capacity compared to rtM204I mutation.

## Introduction

Infection of human hepatitis B virus (HBV) has been a long-standing public health burden with an estimation of 240 million infections worldwide by July 2012 [1]. Interferon and nucleos(t)ide analogs (NA) have been applied for management of HBV infection. NA inhibits the viral replication through competing for incorporation into viral DNA and has largely attenuated the hazardous outcome of HBV infection despite of poor off-treatment responses, failure of cccDNA eradication [2], [3]. Prolonged NA treatment increased the chance of drug resistance, which could lead to treatment failure. Drug resistance related virologic breakthrough has been defined as increase in serum HBV DNA by 1 log (10-fold) after achieving virologic responses [4].

Four NAs including lamivudine (LAM), adefovir dipivoxil (ADV), telbivudine (LdT), and entecavir (ETV) have been officially approved for clinical use by China Food and Drug Administration, while tenofovir dipivoxil (TDF) is in clinical trail phase III in China. LAM is still widely used in single or in combination in practice in China mostly due to financial reasons [5], [6]. However, the disadvantages of LAM are obvious: it is prone to select drug resistant mutation with the highest resistance rate for all known NAs. The reported annually resistance mutation rate was 14–32% and was over 60–70% after 48 months of treatment [4], [7]. Signature LAM resistance mutations have been mapped to the tyrosine-methionine-aspartate-aspartate (YMDD) locus in the catalytic or C domain of HBV reverse transcriptase (RT) region [8], [9], and the primary resistance mutations are isoleucine (I), valine (V), or occasionally serine (S) that were designated rtM204I/V/S. The rtM204I also confers cross-resistance to LdT. Compensatory mutations rtL180M, rtV173L, and rtL80I to LAM are often co-selected to restore viral replication efficacy [10]. Resistance to LAM has been studied extensively, but the full picture of LAM resistance is far from clear. Close surveillance for drug resistance was strongly suggested during NA treatment, while it was complicated by substitutions outside the well-defined positions [11]. Even at the same position, it is not clear whether all forms of substitution would cause drug resistance. This study aimed to clarify whether a newly-found rtM204Q mutant from patients was associated with drug resistance.

## Methods

### Patients and materials

A total of 12,000 serum samples from 9,830 chronically HBV-infected patients who visited Beijing 302 Hospital (from April 2007 to September 2011) were screened in the study. The illness categories include chronic hepatitis B and HBV-related liver cirrhosis. All patients had received various schedules of NA treatment. The diagnostic criteria were based on 2000 Xi'an Viral Hepatitis Management Scheme issued by the Chinese Society of Infectious Diseases and Parasitology, and the Chinese Society of Hepatology, of the Chinese Medical Association [12], and had been described in details in our previous studies [13], [14], [15]. Patients who were co-infected with other hepatitis viruses or human immunodeficiency virus were excluded. This study was approved by the Ethics Committee of Beijing 302 Hospital, and written informed consent was obtained from all participants.

### Serological markers, quantitation of HBV DNA and sequencing of HBV RT gene

Biochemical and serological markers and HBV DNA level of the patients were routinely detected in the Central Clinical Laboratory of Beijing 302 Hospital. HBV DNA level was determined using a real-time quantitative PCR kit (Fosun Pharmaceutical Co., Ltd., Shanghai, ) with a lower detection limit of 100 IU/mL. HBV DNA was extracted from patient's serum by DNAout (Tianenze, Beijing, China) and the RT-region (nt 54–1278) was subjected to an in-house PCR with a sensitivity of 20 IU/mL (Chinese patent ZL 200910092331.1) as previously described [14]. The sense and antisense primers for the first-round PCR were 5′-AGTCAGGAAGACAGCCTACTCC-3′ (nt 3146–3167) and 5′-AGGTGAAGCGAAGTGCACAC-3′ (nt 1577–1596), respectively. The sense and antisense primers for the second-round PCR were 5′-TTCCTGCTGGT-GGCTCCAGTTC-3′ (nt 54–75) and 5′-TTCCGCAGTAT-GGATCGGCAG-3′ (nt 1258–1278), respectively. The PCR products were directly sequenced for all patients. Clonal sequencing for interested patients was performed (≥20 clones per sample) at the time of virologic breakthrough. The sequences of representative HBV RT genes from each rtM204Q-positive patient were submitted to GenBank.

### Construction of viral amplicons containing 1.1mer HBV genome

Amplicons containing 1.1mer genotype C HBV genome which harbored naturally-occurred rtM204Q, rtM204I, rtA181T, rtA181T+M204Q mutant RT genes or wild-type RT gene from the same patient were constructed for phenotyping of drug resistance based on pTriEx-mod-1.1 vector which was a kind gift by professor Zoulim [16]. Briefly, the HBV polymerase mutants identified by the clonal sequencing were inserted in the 1.1mer HBV genome by replacing the 1109 bp *Xho* I-*Sph* I fragment of the vector containing HBV with the corresponding *Xho* I-*Sph* I fragment from selected pGEMT-easy HBV polymerase plasmids. The obtained constructs were verified by sequencing analysis.

### Assessment of viral replication capacity and drug susceptibility

Viral replication capacity and drug susceptibility were evaluated as previously described with minor modification [17]. Briefly, the constructs were transiently transfected into HepG2 cells before the adding of serially diluted NAs. HepG2 cells were firstly cultured in 24-well plates with 1×10^5^ cells per well before transfection. Then every well was transfected with 0.25 µg plasmids by FuGene HD transfection reagent (Roche, Mannheim, Germany) and the transfection efficiency was normalized by β-galactosidase with reporter plasmid (Promega, Madison, WI, USA). Four hours after the transfection, serially diluted NA (LAM and TDF: 0, 0.01, 0.1, 1.0, 10, and 100 µmol/L; ADV: 0, 0.033, 0.1, 0.33, 1.0, and 3.3 µmol/L; ETV: 0, 0.001, 0.01, 0.1, 1.0, and 10 µmol/L) was applied to the transfected HepG2 cells every two days. Four days after cultivation, cells were harvested and lysed. Viral core particles were pulled down using anti-HBc/protein A+G. HBV replicative intermediates in core particle were released and quantitated by real-time PCR. The experiments were performed at least 3 times independently.

### Statistical analysis

Data are presented as mean ± standard deviation or median (range). Differences between variables were examined by Student's *t*-test. Statistical analysis was carried out in Statistical Program for Social Sciences (SPSS 16.0 for Windows; SPSS Inc., Chicago, IL). A *P* value of <0.05 (2-tailed) was considered statistically significant.

## Results

### Drug resistance mutation profile and clinical data of patients

The rtM204Q mutation of RT domain was detected in seven LAM-refractory and three LdT-refractory patients by direct sequencing, and the detection rate of rtM204Q among the chronically HBV-infected patients in Beijing 302 Hospital was 0.1% (10/9,830). By contrast, rtM204I/rtM204V was detected in 2,502 patients with an incidence of 25.5% (2,502/9,830). The rtM204Q emerged either alone or in concomitance with other mutations (rtM204I/V, rtA181T, rtL180M, rtT184I) by direct sequencing. All patients detected with rtM204Q were infected with genotype C, serotype *adr* HBV and had a moderate to high level of HBV viral load (DNA >10^4^ IU/mL) when antiviral therapy initiated. Seven were receiving LAM at the time of the testing. The median duration for LAM therapy to refractory was 28 months (range: 17–35). The substitution information and clinical data for the seven patients were summarized in Table 1. Fourteen representative HBV RT sequences from each rtM204Q-positive patient were deposited in GenBank (accession number KJ011529 through KJ011542).

**Table 1:** Mutations by direct sequencing and clinical data of the studied patients.

	Age yrs	HBV DNA Lg IU/mL	ALT U/L	HBeAg	Genotype/serotype	NA history (months)	Virologic breakthrough	Mutational pattern (codon change for M204Q)
Patient 1	27	6.27	126	(−)	C/adr	LAM (17)	Yes	rtM204Q/I+A181T (ATG→CAA)
Patient 3	34	2.57	62	(−)	C/adr	LAM (31)	Yes	rtM204Q/V (ATG→CAG/A)
Patient 4	46	3.96	18	(+)	C/adr	LAM (24)	Yes	rtM204Q (ATG→CAG)
Patient 6	37	2.88	23	(+)	C/adr	LAM (28)	Yes	rtM204Q (ATG→CAG)
Patient 8	38	2.63	21	(−)	C/adr	LAM (35)	Yes	rtM204Q (ATG→CAA)
Patient 9	52	2.37	20	(+)	C/adr	LAM (30)	Yes	rtM204V/Q+L180M+T184I(ATG→CAG)
Patient 10	46	2.47	16	(−)	C/adr	LAM (23)	Yes	rtM204Q/I (ATG→CAA)

### Clinical course and clonal analysis of three rtM204Q-positive patients during antiviral treatment

Drug-resistant mutant dynamics during the clinical course were analyzed for patient 1, patient 4, and patient 6. All three patients harbored overwhelming (100%) wild-type viral strains in the baseline samples (Sa1, Sb1, Sc1) by clonal sequencing (20 clones for each sample). The patients responded well to LAM before virologic breakthrough. rtM204Q mutant was predominantly detected in the patients' samples collected at the time-point of virologic breakthrough.

**Figure 1:**
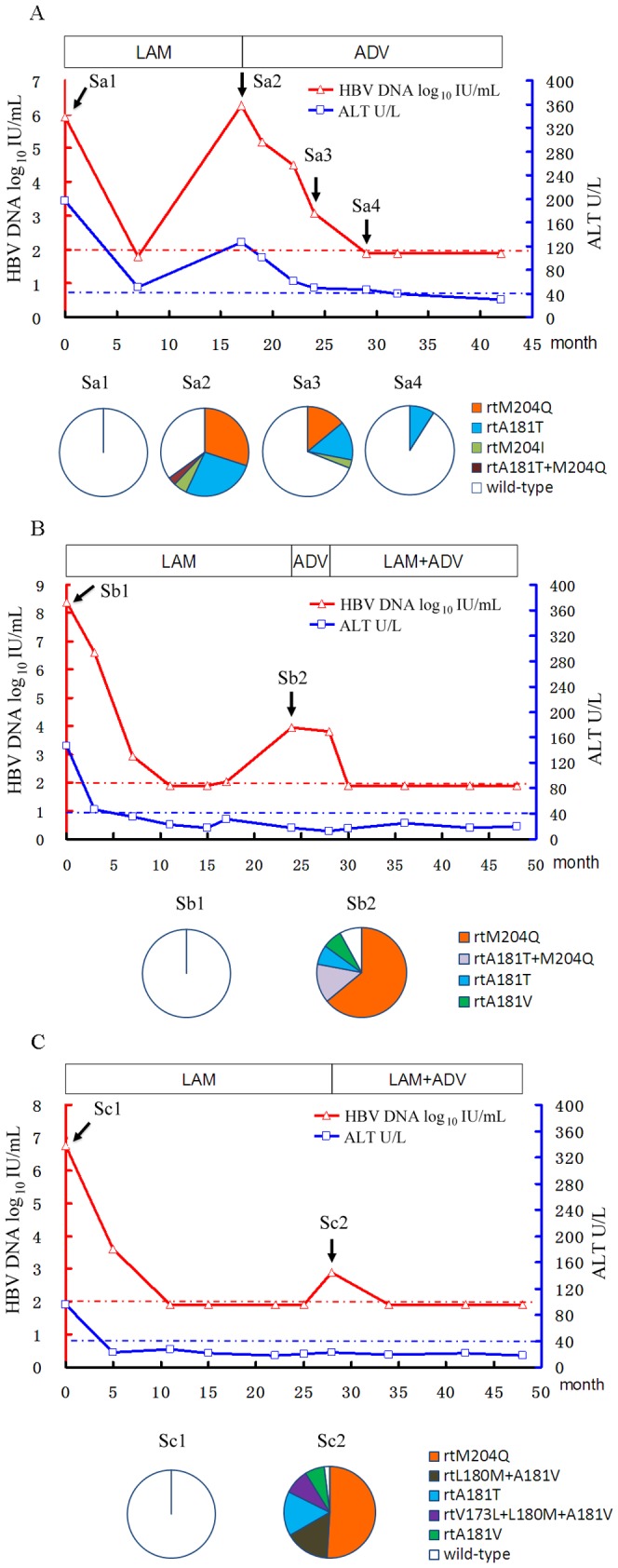
Follow-up the clinical course of three representative patients with HBV rtM204Q mutant during antiviral treatment. The dynamic changes of serum HBV DNA and alanine aminotransferase (ALT) levels are shown along with the successive antiviral therapies for patient 1 (A), patient 4 (B), and patient 6 (C). The duration (months) of the therapies for antivirals is indicated by bars and the successfully-sequenced samples are indicated by arrows with sample (S) numbers above the graph. The dashed red and blue lines show the lower detection limit of HBV DNA and normal ALT levels, respectively. Proportions of wild-type and mutant strains in the viral reverse transcriptase domain of each sample are depicted by a series of pie-charts. ADV, adefovir dipivoxil; LAM, lamivudine.

Patient 1 started LAM treatment with serum HBV DNA of 5.92 log_10_ IU/mL and alanine aminotransferase (ALT) level of 197 U/L. After seven months treatment, serum HBV DNA and ALT levels reduced to 1.8 log_10_ IU/mL and 51 U/L, respectively. At month 17, serum HBV DNA and ALT levels increased to 6.27 log_10_ IU/mL and 126 U/L, respectively. Clonal sequencing (37 clones) of the sample at this point (Sa2) showed coexistence of rtM204Q (30%), rtA181T (27%), rtM204I (5%), rtA181T+M204Q (3%) and wild-type (35%) strains in the viral pool. Then the treatment was switched to ADV, which reduced HBV DNA and ALT levels gradually. Clonal sequencing (35 clones) of the sample at months 24 (Sa3) showed that rtM204Q, rtA181T, rtM204I, rtA181T+M204Q and wild-type strains occupied 14%, 14%, 3%, 0%, and 69%, respectively. Clonal sequencing (35 clones) of the sample at month 29 (Sa4) showed 91% wild-type strain and 9% rtA181T mutant in the viral pool. Afterwards, HBV DNA remained undetectable with persistent normal ALT in the next 13 months following-up (Figure 1A).

Patient 4 received LAM treatment with serum HBV DNA of 8.37 log_10_ IU/mL and ALT level of 147 U/L. The treatment was effective before virologic breakthrough occurred at month 24. Clonal sequencing (28 clones) of the sample at this point (Sb2) showed predominance of rtM204Q strain (64%) concomitant with rtA181T+M204Q (14%), rtA181T (7%), rtA181V (7%) and wild-type (7%) strains in the viral pool. Afterwards, the treatment was switched to ADV for 4 months and then adapted to LAM plus ADV because of non-response to single ADV treatment. The combination therapy effectively reduced serum HBV DNA to undetectable level in two months (at month 30). Viral gene failed to be amplified for sequence analysis from the samples collected at this point and subsequent point. The patient showed good virologic and biochemical responses for the rest 18 months of following-up (Figure 1B).

Patient 6 started LAM treatment with serum HBV DNA of 6.77 log_10_ IU/mL and ALT level of 96 U/L. The treatment was effective before virologic breakthrough occurred at month 28. Clonal sequencing (44 clones) of the sample at this point (Sc2) showed predominance of rtM204Q strain (52%) concomitant with rtL180M+A181V (16%), rtA181T (16%), rtV173L+L180M+A181V (9%), rtA181V (5%) and wild-type (2%) strains in the viral pool. ADV adding-on to LAM was taken as a rescue therapy, which effectively reduced serum HBV DNA to undetectable level in two months (at month 34). Viral gene failed to be amplified for sequence analysis from the samples collected at this point and subsequent point. The patient showed good virologic and biochemical responses for the rest 14 months of following-up (Figure 1C).

### Replication capacity and drug resistance of rtM204Q

**Figure 2:**
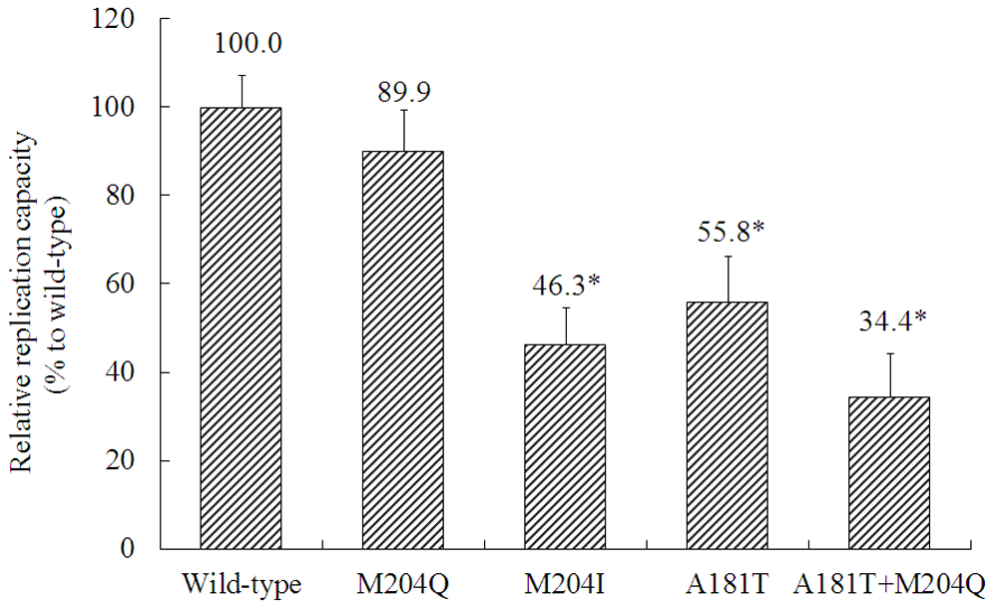
Assessment of HBV natural replication capacity of HBV strains isolated from patient 1. The relative replication capacities of mutants were analyzed compared to that of wild-type strain (defined as 100%) in the absence of drug. Data are presented as mean ± standard deviation. Experiments were performed at least 3 times independently. *P<0.05 (*vs*. wild-type strain).

Phenotypic analysis of drug resistance was performed for HBV mutants from patient 1. The results showed that rtM204Q mutant had a replication capacity intermediate to that of rtM204I mutant and wild-type HBV, while rtA181T+M204Q had the lowest natural replication capacity among the wild-type HBV and the four tested mutants (Figure 2). Phenotypic analysis of drug resistance showed that rtM204Q and rtM204I mutants had 76-fold and 1396-fold increased LAM EC_50_ of the wild-type strain respectively, and both of the mutants remained susceptible to ADV, ETV and TDF compared to the wild-type strain. The results of drug susceptibility of different mutants are shown in Table 2.

**Table 2:** Drug susceptibility of rtM204Q and rtM204I mutants and wild-type strain.

	Wild-type		rtM204Q		rtM204I	
	EC50	fold	EC50	fold	EC50	fold
Lamivudine	0.158±0.012	1.00	11.96±2.36	75.68	220.50±29.85	1395.57
Adefovir	0.089±0.008	1.00	0.168±0.092	1.89	0.085±0.021	0.96
Entecavir	0.00196±0.0002	1.00	0.00033±0.00009	0.17	0.0068±0.0009	3.47
Tenofovir	0.024±0.005	1.00	0.113±0.020	4.69	0.072±0.008	2.98

EC_50_, 50% effective concentration of drug (µ mol/L).

## Discussion

Viral resistance mutations have several distinguishing characteristics, including (1) an association with virologic breakthrough during the drug therapy; (2) appearance in multiple patients exposed to the drug; (3) ability to confer resistance to the virus *in vitro*; and (4) reversion to wild-type sequence in the absence of the selective antiviral pressure due to reduced replication capacity of the mutant compared to the wild-type virus [18]. In this study, we reported a novel LAM-resistance-associated mutation rtM204Q after screening of a large number of patients with chronic HBV infection. Accordingly, rtM204Q was detected in seven LAM-refractory patients and the emergence of the mutation was closely linked with virologic breakthrough in clinical course for all of the seven patients and biochemical breakthrough in two of them. rtM204Q mutant was proved being predominant in three representative patients' samples by clonal sequencing at the time of virologic breakthrough. In addition, after rescue therapy with ADV or ADV plus LAM, proportion of rtM204Q mutant obviously declined and wild-type strain obviously increased simultaneously with the decrease of viral load as shown in patient 1. Phenotypic analysis of drug resistance showed that the rtM204Q mutation significantly reduced LAM susceptibility of the mutant virus. The evidence together suggested rtM204Q as a main contributor for the drug resistance of the LAM-refractory patients.

For the position of rt204 in the HBV RT region, rtM204V and rtM204I are well-known primary LAM- and/or LdT-resistant mutation [19], [20], and rtM204S was also reported to be induced by LAM treatment and conferred LAM resistance [21]. The position 184 in RT domain of human immunodeficiency virus shared similar profile of resistance pattern to LAM, such that rtM184I appears early in therapy, and rtM184V replaces rtM184I later, the outgrowth of rtM184V was a balance between mutation and selection [22]. Molecular modeling had suggested that the YMDD motif was critical in geometric alignment for base pairing of the incoming dNTP with the template [23], [24]. Study on hypothetical mutation patterns at position rt204 of HBV elucidated that mutants with substitution (I, V, A, and L) at rt204 may still replication-competent, mutants with V and I substitution reduced sensitivity to LAM, and one amino acid substitution at rt204 was potential to cause LAM resistance *in vitro* [25]. The rtM204K mutation was reported to be found in a LAM unsuccessfully-treated patient [26]. A previous study reported the emergence of rtM204Q with rtA181T in a TDF-treated ADV-resistant patient [27]. We also found the emergence of rtM204Q in one case refractory to LAM [28]. However, the clinical incidence and association of rtM204Q mutation with LAM resistance remained unclear. In this study, three forms of mutation at rt204 were detected resistant to LAM in this study, namely V, I and Q. The rtM204I and rtM204V were the mostly detected mutations and were found in 25.5% of the patient population and rtM204Q was only found in 0.1% of the patient population. The rtM204I mutation only requires one nucleotide change, i.e., from ATG to ATT, ATA, or ATC. The rtM204V usually requires one nucleotide change, i.e., from ATG to GTG, and infrequently requires two nucleotide change, i.e., from ATG to GTT/GTC/GTA. By contrast, rtM204Q mutation requires two or three nucleotide changes (from ATG to CAG or CAA), which increases genetic barrier of the mutation and could be one reason for its low incidence in clinic.

Viral fitness is a key factor associated with the development of antiviral resistance. Viral fitness generally refers to the ability of a virus to replicate in a defined environment. Usually, mutant viruses are “less fit”, meaning they do not replicate as well as wild-type virus, but may have a survival advantage in the presence of an antiviral agent [29]. In fact, both drug susceptibility and natural replication capacity affected viral fitness. Phenotypic analysis showed that rtM204Q mutant was less resistance to LAM compared with rtM204I mutant (of 76-fold vs. 1395-fold that of the wild-type strain), but it had higher natural replication capacity compared with rtM204I (of 89.9% vs. 46.3% that of the wild-type strain). This may account for that rtM204Q had competence over rtM204I mutant and wild-type strains in a few of cases. In addition to drug pressure, host immune pressure is another factor affecting viral fitness [30]. The resistant mutations may alter overlapping S-gene coding and influence anti-S immune response or introduce effective trans-complementation of viral mutants [31], [32]. Moreover, a recent analysis of Bayesian networks that connect rtM204I/V to many sites of HBV proteins confirmed that LAM resistance was a complex trait encoded by the entire HBV genome rather than by a single mutation [33]. Whether these factors affect development of rtM204Q mutation needs further study.

The rtA181T mutant was simultaneously detected with rtM204Q in patient 1, patient 4 and patient 6; and rtA181V mutant was simultaneously detected with rtM204Q in patient 4 and patient 6. The rtA181T has been related with LAM exposure and it induces a stop codon (sW172stop) in the overlapping hepatitis B surface protein [34]. The rtA181V as a primary mutation of ADV resistance could also be induced by LAM though it was not a primary mutation of LAM [35], [36]. Patient 4 had poor response to ADV rescue therapy but had good response to subsequent combined therapy of LAM plus ADV. This could be associated with rtA181V mutant in the patients.

The rtM204Q mutant remained susceptible to ETV, ADV, and TDF in phenotypic analysis. This suggested that rtM204Q mutation, like rtM204I and rtM204V mutations, was specific to LAM resistance. In line with the *in vitro* results, clinical switching-to or combination with ADV effectively suppressed viral replication of these patients.

In conclusion, our data showed that rtM204Q confers a significant decreased susceptibility to LAM with relatively competent replication capacity and associates with virologic breakthrough during LAM treatment. Therefore, rtM204Q might be a novel LAM-resistance-associated mutation. Switching-to or adding-on of ADV could serve as a rescue therapy for rtM204Q related LAM refractory patients.
